# Stroke as the First Manifestation of Atrial Fibrillation

**DOI:** 10.1371/journal.pone.0168010

**Published:** 2016-12-09

**Authors:** Jussi Jaakkola, Pirjo Mustonen, Tuomas Kiviniemi, Juha E. K. Hartikainen, Antti Palomäki, Päivi Hartikainen, Ilpo Nuotio, Antti Ylitalo, K. E. Juhani Airaksinen

**Affiliations:** 1 Heart Center, Turku University Hospital and University of Turku, Turku, Finland; 2 Department of Medicine, Keski-Suomi Central Hospital, Jyväskylä, Finland; 3 Heart Center, Kuopio University Hospital, Kuopio, Finland; 4 Neurocenter, Neurology, Kuopio University Hospital, Kuopio, Finland; 5 Division of Medicine, Department of Acute Internal Medicine, Turku University Hospital, Turku, Finland; 6 Heart Center, Satakunta Central Hospital, Pori, Finland; University of Minnesota, UNITED STATES

## Abstract

Atrial fibrillation may remain undiagnosed until an ischemic stroke occurs. In this retrospective cohort study we assessed the prevalence of ischemic stroke or transient ischemic attack as the first manifestation of atrial fibrillation in 3,623 patients treated for their first ever stroke or transient ischemic attack during 2003–2012. Two groups were formed: patients with a history of atrial fibrillation and patients with new atrial fibrillation diagnosed during hospitalization for stroke or transient ischemic attack. A control group of 781 patients with intracranial hemorrhage was compiled similarly to explore causality between new atrial fibrillation and stroke. The median age of the patients was 78.3 [13.0] years and 2,009 (55.5%) were women. New atrial fibrillation was diagnosed in 753 (20.8%) patients with stroke or transient ischemic attack, compared to 15 (1.9%) with intracranial hemorrhage. Younger age and no history of coronary artery disease or other vascular diseases, heart failure, or hypertension were the independent predictors of new atrial fibrillation detected concomitantly with an ischemic event. Thus, ischemic stroke was the first clinical manifestation of atrial fibrillation in 37% of younger (<75 years) patients with no history of cardiovascular diseases. In conclusion, atrial fibrillation is too often diagnosed only after an ischemic stroke has occurred, especially in middle-aged healthy individuals. New atrial fibrillation seems to be predominantly the cause of the ischemic stroke and not triggered by the acute cerebrovascular event.

## Introduction

Atrial fibrillation (AF) is a major risk factor of thromboembolism and ischemic stroke [1], However, the risk is effectively diminished by oral anticoagulation therapy [2]. The arrhythmia is often asymptomatic or ‘silent’[3–5], but the risk of ischemic stroke seems to be similar regardless of the presence or absence of symptoms[3]. Furthermore, approximately 1/3 of all ischemic strokes are of an unknown cause [6] and it is often speculated that silent paroxysmal AF also lies behind many of these cryptogenic strokes. Recent studies support this notion and have shown that more diligent monitoring of heart rhythm with ambulatory devices after a cryptogenic stroke uncovers a high number of silent AF episodes[7–8]. Asymptomatic AF poses a difficult diagnostic challenge to clinicians. These patients are more likely to evade diagnostic effort and without appropriate anticoagulation they are left vulnerable to thromboembolism and ischemic stroke. The aim of this study was to assess how often an ischemic stroke or a transient ischemic attack (TIA) is the first clinical manifestation of AF in a cohort of patients with diagnoses of both AF and ischemic stroke or TIA.

## Materials and Methods

This study is a pre-specified analysis of the multicenter retrospective FibStroke Study (ClinicalTrials.gov Identifier: NCT02146040) assessing thromboembolic complications and intracranial hemorrhages in patients with AF[9–10]. It is also a part of ongoing wider protocol in Western Finland to evaluate thromboembolic and bleeding complications of AF[11–13].

All patients over the age of 18 who were diagnosed with AF at any time and with an ischemic stroke, a TIA, or an intracranial hemorrhage during 2003–2012 were retrospectively identified from the institutional discharge registries and clinical records of two university hospitals (Turku University Hospital, Turku; Kuopio University Hospital, Kuopio) and two central hospitals (Satakunta Central Hospital, Pori; Keski-Suomi Central Hospital, Jyväskylä), which were responsible for the treatment of all stroke and TIA patients in their respective regions during the study period. After identification, each case was individually reviewed from their patient records with a standardized data collection protocol. Information was recorded on patient characteristics, risk factors of stroke or intracranial bleeding, and heart rhythm at the time of the ischemic or bleeding event. The use of medication, major operations, cardioversions, and bleeding events during the preceding period of 30 days were also reviewed. The patients were further followed from the patient records for 30 days after the ischemic or bleeding event to evaluate all-cause mortality.

First, a total of 5,885 ischemic events (4,547 of which were strokes and 1,338 TIAs) were identified in 5,039 patients. In the primary analysis, only the first ever ischemic events of patients in whom AF was diagnosed before or during the hospitalization for the event were included. Thus, the primary study group consisted of 3,623 ischemic events (2,914 strokes and 709 TIAs) in 3623 patients (S1 Fig). These patients were then divided into two groups according to the time of AF diagnosis: 1) patients with a history of AF before the first ischemic stroke or TIA designated as ‘previous AF’ and 2) patients with the diagnosis of AF made during the index hospitalization (i.e. detected at the presentation of ischemic stroke or TIA in the emergency room or within the following week) designated as ‘new AF’.

To uncover potential clustering of AF diagnoses around the first ischemic event, we performed a supplementary analysis including also 588 patients in whom AF was diagnosed after hospitalization for their first ever ischemic event. This analysis focused on AF diagnoses made within 8–30 days after hospitalization for the ischemic event, since these late diagnoses may also reflect the potential role of silent paroxysmal AF as the cause of the index ischemic event.

To elucidate the role of cerebrovascular events as a trigger of new arrhythmias we compiled a control group of 781 patients with intracranial hemorrhages according to the same exclusion criteria used with the ischemic events. These patients were divided into previous AF and new AF groups using the same criteria as with the ischemic events.

The diagnosis of AF was confirmed by an electrocardiographic recording according to the standard criteria. An ischemic stroke was defined as a permanent focal neurological deficit adjudicated by a neurologist, and confirmed by computed tomography or magnetic resonance imaging. TIA was defined as a transient (<24 hours) focal neurological deficit adjudicated by a neurologist. Only ischemic strokes and TIAs that were considered definite by the treating physician were included in the present study. Intracranial hemorrhages were adjudicated by a neurologist and confirmed by computed tomography or magnetic resonance imaging.

The study protocol was approved by the Medical Ethics Committee of the Hospital District of Southwest Finland and the ethics committee of the National Institute for Health and Welfare. Informed consent was not required because of the retrospective registry nature of the study. The study conforms to the Declaration of Helsinki.

Statistical analyses were performed with the SPSS software (version 22.0, SPSS, Inc., Chicago, Illinois). Continuous data are presented as median [interquartile range] and categorical variables as absolute number and percentage. Chi-square and Fisher’s exact tests were used to compare differences between proportions. Mann-Whitney U test was used for analysis of continuous variables. Based on the results of bivariable comparisons, logistic regression analysis was performed to analyze the independent predictors of new AF and 30-day mortality after an ischemic stroke. Two-sided differences were considered significant if the null hypothesis could be rejected at the 0.05 probability level.

## Results

The median age of the patients with previous or new AF was 78.3 [13.0] years and 2,009 (55.5%) of them were women. AF was diagnosed prior to the ischemic event in 2,870 (79.2%) patients (previous AF group), and in 753 (20.8%) patients (new AF group) the diagnosis was concurrent with the ischemic event. When analyzed separately, new AF was diagnosed in 637 (21.9%) patients with an ischemic stroke and in 116 (16.4%) patients with a TIA. In the new AF group, AF or atrial flutter was present already on admission in 534 (70.9%) and 21 (2.8%) patients, respectively, and were detected later during the index hospitalization in 198 (26.3%) patients. The proportion of new AF did not change significantly during the course of the study period (p = 0.132). In comparison, new AF was diagnosed in only 15 of the 781 patients (1.9%) with an intracranial hemorrhage.

**Fig 1:**
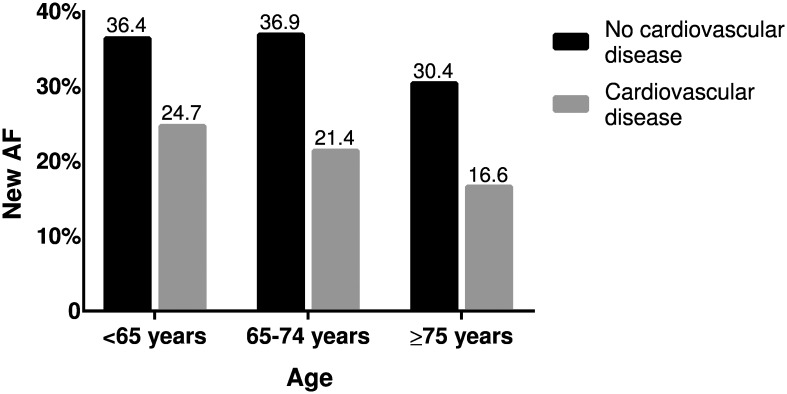
New AF diagnoses at the time of ischemic stroke according to age and cardiovascular diseases. Cardiovascular diseases include coronary artery disease, other vascular diseases, congestive heart failure and hypertension. Cardiovascular disease: N = 2,271 (<65 years: N = 215; 65–74 years: N = 533; ≥75 years: N = 1,523). No cardiovascular disease: N = 643 (<65 years: N = 107; 65–74 years: N = 187; ≥75 years: N = 349). Abbreviations: AF, atrial fibrillation.

**Fig 2:**
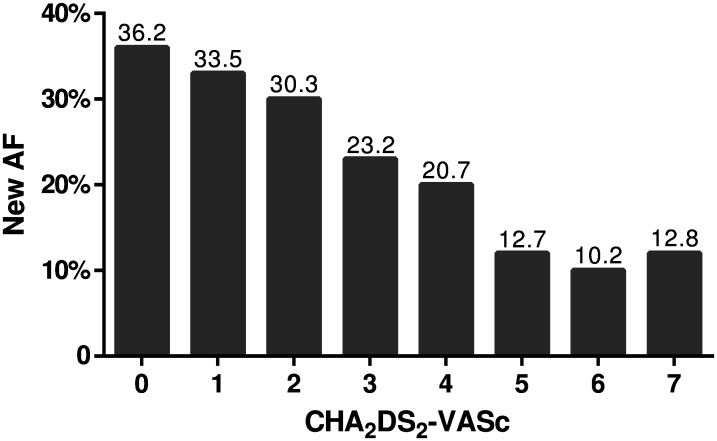
New AF diagnoses at the time of ischemic stroke according to CHA_2_DS_2_-VASc score. CHA_2_DS_2_-VASc score is calculated at the time of the ischemic event and scoring does not include the current event. N = 2,914 (score 0: N = 38; score 1: N = 64; score 2: N = 127; score 3: N = 155; score 4: N = 163; score 5: N = 64; score 6: N = 21; score 7: N = 5). Abbreviations: AF, atrial fibrillation.

Patients with new AF were younger, had a higher heart rate, and a lower CHA_2_DS_2_-VASc score than patients with previous AF (Table 1). Patients with previous AF presented more often with a history of hypertension, diabetes, coronary artery disease, other vascular diseases, heart failure, and myocardial infarction and had underwent more often surgery or other procedures during the 30 days preceding the ischemic event (Table 1). In multivariable logistic regression analysis, the independent predictors of new AF in patients with an AF-associated ischemic event were younger age (odds ratio [OR]: 1.01; 95% confidence interval [CI]: 1.00–1.02; p = 0.014), absence of history of coronary artery disease (OR: 1.45; 95% CI: 1.16–1.82; p = 0.001), other vascular diseases (OR: 1.75, 95% CI: 1.09–2.79. p = 0.020), heart failure (OR: 2.62; 95% CI: 1.94–3.54, p<0.001), or hypertension (OR: 1.22; 95% CI:1.01–1.47; p = 0.041), and high heart rate (OR: 1.02, 95% CI: 1.02–1.03; p<0.001). The proportion of new AF diagnoses at the time of an ischemic stroke according to the patients’ age and history of cardiovascular diseases is presented in Fig 1. Similarly, the proportion of new AF was highest (36.2%) in those ischemic stroke patients with a CHA_2_DS_2_-VASc score of 0 and lowest (12.1%) in those with a score of ≥5 (Fig 2).

**Table 1:** Baseline and clinical characteristics of the patients at the time of the ischemic stroke or TIA.

N	Previous AF 2870	New AF 753	p value
Age, years	78.7 [12.9]	76.6 [13.6]	<0.001
Age groups			<0.001
• <65	333 (11.6)	109 (14.5)	
• 65 to 74	696 (24.3)	219 (29.1)	
• ≥75	1841 (64.1)	425 (56.4)	
Female gender	1592 (55.5)	417 (55.4)	0.967
Heart rate	75.0 [28.0]	94.0 [43.0]	<0.001
Hypertension	1886 (65.8)	442 (58.7)	<0.001
Dyslipidemia	990 (34.7)	238 (31.8)	0.141
Diabetes	607 (21.2)	125 (16.6)	0.006
Coronary artery disease	968 (33.8)	151 (20.1)	<0.001
Previous myocardial infarction	415 (14.5)	58 (7.7)	<0.001
Other vascular diseases	202 (7.0)	23 (3.1)	<0.001
Heart failure	635 (22.1)	66 (8.8)	<0.001
CHA_2_DS_2-_VASc score	4.0 [2.0]	3.0 [2.0]	<0.001
• 0–1	283 (9.9)	120 (15.9)	
• 2	386 (13.4)	151 (20.1)	<0.001
• 3–5	1948 (67.9)	449 (59.6)	
• >5	253 (8.8)	33 (4.4)	
Surgery or other procedure <30 days prior to event	179 (6.2)	17 (2.3)	<0.001
Cardioversion (electrical or chemical) <30 days prior to event	93 (3.2)	0 (0.0)	<0.001
Type of AF			<0.001
Paroxysmal	1105 (42.6)	474 (87.0)	
Persistent	30 (1.2)	8 (1.5)	
Chronic	1461 (56.3)	63 (11.6)	

Values are presented as median [interquartile range] or number (%).

Abbreviations: AF, atrial fibrillation; TIA, transient ischemic attack.

Ischemic stroke patients with previous AF had a higher 30-day mortality than those with new AF (15.1% vs. 8.7%, p<0.001), and previous AF remained an independent predictor of 30-day mortality even after adjusting for confounding factors (age, gender, dyslipidemia, coronary artery disease, other vascular diseases, and heart failure) (OR 1.44; 95% CI: 1.05–1.97; p = 0.025). Additionally, 6 fatalities occurred in the patients with TIA, all in patients with previous AF.

**Fig 3:**
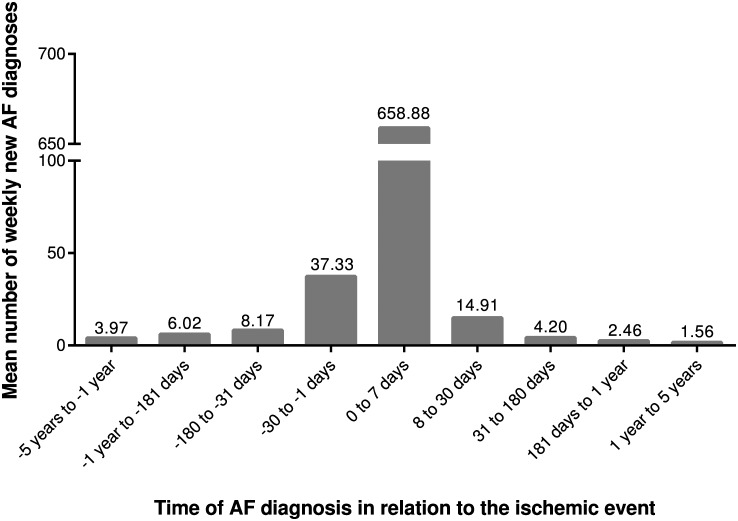
First AF diagnoses according to temporal distance from first ischemic event. The mean number of first AF diagnoses per one week is presented according to temporal distance from the first ischemic event. Ischemic stroke/TIA has occurred at time point zero. Negative values portray time before the event and positive values time after the event. Timing could not be reliably classified in 740 patients with a long history of AF: N = 2,605.

In the supplementary analysis, AF was diagnosed during the first month (excluding the first week of the index hospitalization) after the ischemic event in 49 patients (incidence rate of 14.9/week). During a later follow-up of 5 years (excluding the first month after the event) AF was diagnosed in an additional 481 patients (mean incidence rate of 2.0/week). The mean number of first AF diagnoses per week according to temporal distance from the first ischemic event is depicted in Fig 3.

## Discussion

The current study shows that AF is commonly diagnosed only after an ischemic stroke or TIA has occurred. New AF was found in 21.9% of the patients hospitalized for an ischemic stroke and in 16.4% of the patients hospitalized for a TIA. Remarkably and contrary to current belief, the diagnosis of new AF was most common in younger patients in their working age and free of cardiovascular disease. In the majority (71%) of the new cases, AF was present already on admission. Secondly, there was clustering of new AF diagnoses during the 30-day periods before and after stroke.

The temporal connection between the detection of AF and occurrence of an ischemic stroke has been recognized previously in two small studies. In a report on 115 patients with an AF-associated ischemic stroke, AF was diagnosed during hospitalization for stroke in 26 (22.6%) cases[14]. In another study, AF was diagnosed at the presentation of an ischemic stroke or within 3 days from it in 41 (22.2%) out of 185 strokes[15]. These figures are only slightly higher than in our large contemporary patient cohort and reveal that very little progress has been made in the detection of silent AF despite changes in the management and screening of asymptomatic AF since the 1950s-1990s when these earlier studies were conducted. On the other hand, by effective anticoagulation after the diagnosis of AF, a significant number of strokes are avoided in this population and consequently the proportional share of new, undiagnosed AF in patients with AF-associated stroke should increase. Unfortunately, however, underuse of anticoagulant drugs is still common even in patients with a high CHA_2_DS_2_-VASc score[10].

Central nervous system injury may affect autonomic nervous system and activate a systemic inflammatory response[16–17]. Both of these mechanisms play an important role in the pathogenesis of AF and support the hypothesis that stroke may trigger an acute AF attack[18–19]. One unique feature of our study was to include a “control” group of patients with intracranial hemorrhages to elucidate the direction of causality between AF and intracranial accident. In this setting, we observed that new AF was a seldom finding (1.9%) in patients with intracranial bleeding compared to those with an ischemic event (20.8%). In other words, an intracranial hemorrhage seems to provoke episodes of atrial fibrillation quite rarely supporting the view that the new AF observed during stroke is in most cases the cause of the stroke and not its consequence. On the basis of this novel approach and these observations it seems reasonable to assume that AF was often present either in an asymptomatic or minimally symptomatic form in the majority of the patients with new AF prior to the ischemic event, rather than being precipitated by it, and that thromboembolism was often the causal factor of the stroke. Another finding supporting this assumption is that, in a previous study, in over 90% of patients with newly-diagnosed AF at the presentation of stroke, AF either persisted or recurred after the initial stroke in 24 (92%) of the cases[14].

Another unique feature of our study was that the proportion of new AF diagnoses at the time of an ischemic stroke was investigated also in subgroups deemed particularly susceptible by demographic associations. The size of our patient cohort enabled the characterization of the group of patients in whom AF was diagnosed concurrently with the ischemic event. An unexpected and important finding was that AF was diagnosed during hospitalization for an ischemic stroke in >1/3 of subjects <75 years old or even in their working age and with no history of cardiovascular diseases. Similarly, low CHA_2_DS_2_-VASc scores in ischemic stroke patients were associated with >1/3 of new AF diagnoses during hospitalization. One reasonable explanation for this challenging finding might be that in older people with co-morbidities—and consequently also a high CHA2DS2-VASc score—silent AF may be more easily revealed by health care professionals opportunistically during routine check-up visits. Another conceivable option is that patients with a higher CHA2DS2-VASc score have more shared risk factors for competing etiology of stroke such as carotid atherosclerosis or small artery disease.

Silent AF is not rare in any form: at least 1/3 of all AF is asymptomatic[20]. In the recent EORP-AF study, AF was asymptomatic in 40% of the patients and of those with symptoms more than half had only mild symptoms[21]. Moreover, even in symptomatic patients with paroxysmal AF, the majority of episodes are asymptomatic[22]. The prevalence of silent AF seems to be exceptionally high in patients with pacemaker therapy. Healey et al. recently used implanted cardiac rhythm devices in 2,580 patients over the age of 65 with no previous history of AF and could uncover silent atrial high rate episodes in 34.7% of the patients during a mean of 2.5 years follow-up[3]. The actual prevalence of silent, unrecognized AF at general population level is, however, largely uncharted. Engdahl et al. employed a stepwise screening procedure in the general 75–76 year-old population and found new AF in 4.7% of the study population bringing the total prevalence of AF to 14%[4]. Our LietoAF Study used a simple method based on self-palpation of pulse and observed new AF in 2% of an elderly population already during the first month of the program[23].

Unrecognized AF has long been suspected to lie behind a significant proportion of cryptogenic strokes, and two recent studies utilizing comprehensive screening methods after stroke support the view. CRYSTAL-AF was a randomized controlled study on 441 subjects with cryptogenic stroke that compared implantable cardiac monitoring to standard care in the diagnosis of AF and uncovered new AF in 8.9% after 6 months and 12.4% after 12 months of follow-up in the monitoring arm[7]. Another randomized trial in 572 subjects with cryptogenic stroke, found new AF in 16.1% of the patients with a 30-day event triggered cardiac monitor and in 3.2% of the patients with a 24 hour cardiac monitor[8]. In line with these studies, we found that the incidence rate of first AF diagnoses was elevated during the month after the stroke compared with the incidence rate in later follow-up (Fig 3), and asymptomatic paroxysmal AF might lie behind many of these cryptogenic ischemic events.

Asymptomatic AF carries a 2.5 fold risk of ischemic stroke[3]. The mechanism, however, has recently been questioned by evidence from the ASSERT Investigators, who found no clear temporal association between asymptomatic short AF episodes and stroke[24]. This implies that thromboembolism may not be the only mechanism by which AF causes stroke but also be a general marker of elevated risk of stroke. Nevertheless, the significance of AF as a causative factor of stroke has remained unchanged, which is in agreement with our finding of new AF being a common finding in the context of an ischemic stroke but a seldom one in the context of an intracranial hemorrhage.

Some limitations need to be addressed. The retrospective nature of analyzing patient records is a limitation, but undoubtedly better represents ‘real-world’ practice. Moreover, the completeness and accuracy of the Finnish hospital discharge registry data has been validated previously [25]. When calculating the incidence rates of new AF diagnoses, the timing of AF could not be evaluated reliably in some cases. However, the missing information pertains only to cases where the AF diagnosis was made in the distant past in relation to the ischemic event.

The present findings based on contemporary clinical practice show that AF is too often diagnosed only after an ischemic stroke has occurred, especially in the younger and healthier population. Our findings reinforce the importance of the need for introduction of new and more effective strategies for screening of silent AF.

## Supporting Information

S1 Checklist STROBE checklist for cohort studies. (DOC)

S1 Dataset The underlying relevant data of the study. (XLSX)

S1 Fig Flow chart of the selection and exclusion process of study patients. (TIF)
