# Supplementary figures and images for: Stroke as the First Manifestation of Atrial Fibrillation
Source: PLoS One. 2016 Dec 9;11(12):e0168010. doi: 10.1371/journal.pone.0168010 (PMC5148080; doi:10.1371/journal.pone.0168010)

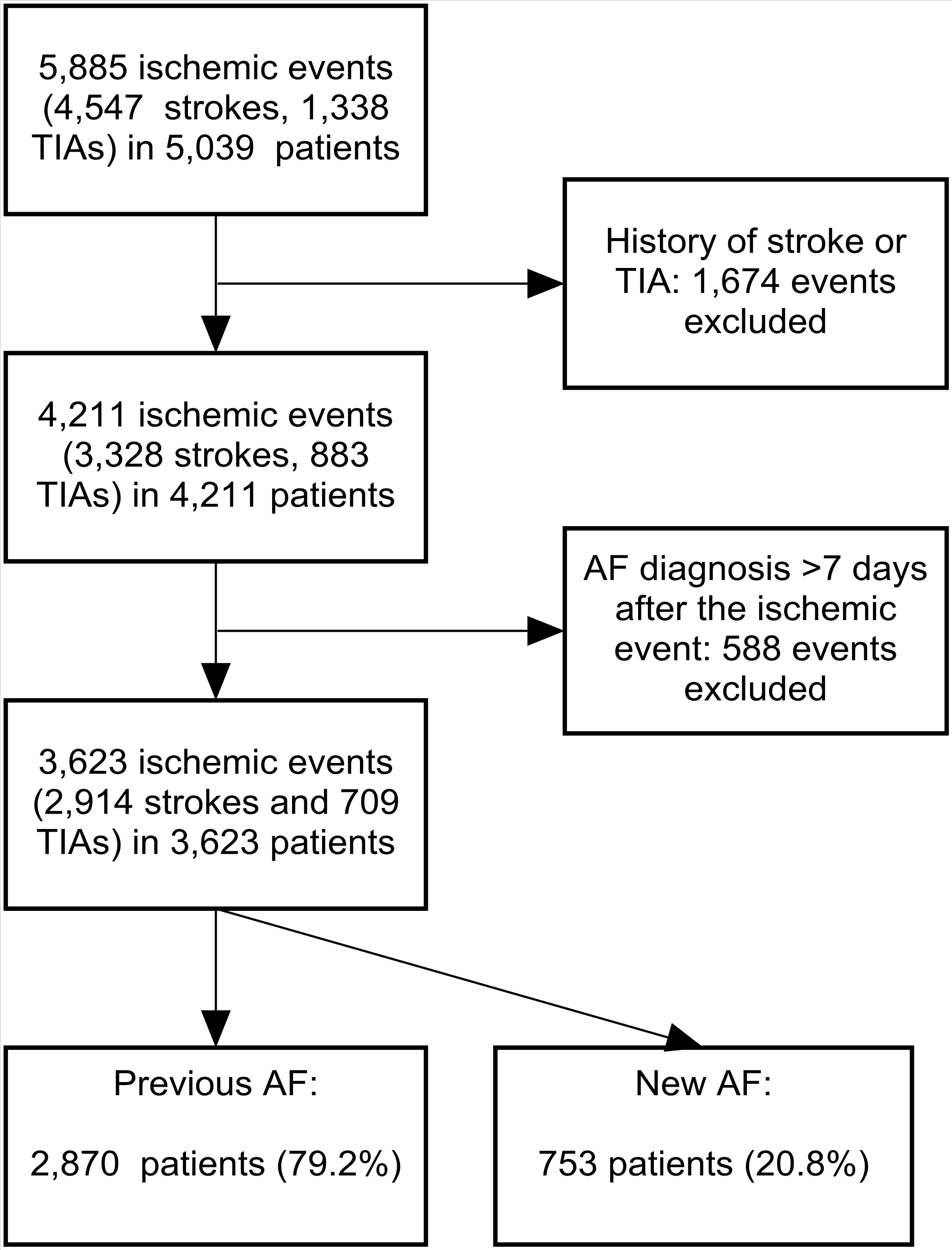

Supplement: S1 Fig — (TIF) [file pone.0168010.s003.tif]
